# Epithelial-Mesenchymal Transition in Pancreatic Carcinoma

**DOI:** 10.3390/cancers2042058

**Published:** 2010-12-09

**Authors:** Harald J. Maier, Thomas Wirth, Hartmut Beug

**Affiliations:** 1 Institute of Physiological Chemistry, University of Ulm, 89081 Ulm, Germany; E-Mail: thomas.wirth@uni-ulm.de; 2 Institute of Molecular Pathology, 1030 Vienna, Austria; E-Mail: hartmut.beug@imp.ac.at

**Keywords:** pancreatic adenocarcinoma, epithelial-mesenchymal transition, tumor progression, cancer stem cells, chemoresistance

## Abstract

Pancreatic carcinoma is the fourth-leading cause of cancer death and is characterized by early invasion and metastasis. The developmental program of epithelial-mesenchymal transition (EMT) is of potential importance for this rapid tumor progression. During EMT, tumor cells lose their epithelial characteristics and gain properties of mesenchymal cells, such as enhanced motility and invasive features. This review will discuss recent findings pertinent to EMT in pancreatic carcinoma. Evidence for and molecular characteristics of EMT in pancreatic carcinoma will be outlined, as well as the connection of EMT to related topics, e.g., cancer stem cells and drug resistance.

## 1. Introduction

### 1.1. Epithelial-Mesenchymal Transition in Tumor Progression

While most malignant tumors are carcinomas, *i.e.*, originate from epithelial tissue, it has long been acknowledged that invading and metastasizing cells from these tumors may share characteristics of mesenchymal cells, suggesting the existence of a transition from an epithelial to a mesenchymal cell phenotype during cancer progression [1].

**Figure 1:**
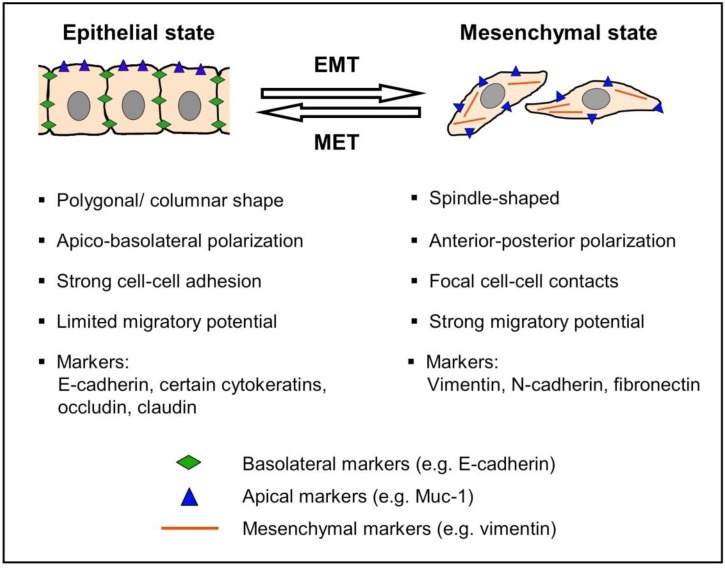
Characteristics of epithelial-mesenchymal transition. EMT: epithelial-mesenchymal transition, MET: mesenchymal-epithelial transition.

Epithelial cells show certain traits that distinguish them from other cell types: They appear columnar or polygonal, display apico-basolateral polarization, and are organized in cell layers with strong cell-cell adhesion. Their migratory potential is limited and confined to movement within the epithelial cell sheet. In contrast, mesenchymal cells are spindle-shaped, exhibit anterior-posterior polarity, grow in a three-dimensional matrix with only focal cell-cell contacts, and exhibit a strong migratory potential. During the process of epithelial-mesenchymal transition (EMT), cells can change from an epithelial to a mesenchymal state: They lose their characteristic epithelial traits and instead gain properties of mesenchymal cells. The molecular correlate of this transition consists in the loss of epithelial markers such as E-cadherin, certain cytokeratins, occludin, and claudin, and the gain of mesenchymal markers such as N-cadherin, vimentin, and fibronectin. After the transition to a mesenchymal state, cells can also change back to an epithelial state in a process known as mesenchymal-epithelial transition (MET; Figure 1) [2].

EMT is a well-established concept in embryonic development and considered essential for mesoderm formation. In recent years, it has been increasingly acknowledged that EMT is also observed in pathological processes, particularly in wound healing, tissue regeneration, organ fibrosis, and tumor progression. While the observed types of EMTs share many common features, a clear distinction should be made between EMTs occurring in different contexts. A classification into three types of EMT has been proposed [2]: Type 1 EMT is used during development to generate cells with mesenchymal features out of epithelial cells. It is a “clean” and entirely physiological process and not associated with inflammation, fibrosis, or an invasive phenotype. Type 2 EMT, in contrast, occurs during tissue repair in response to traumatic or inflammatory injury. Under normal circumstances, type 2 EMT is limited to an acute repair process (e.g., wound healing) and can be beneficial, as it provides tissue replacement. However, if inflammatory stimuli persist, ongoing type 2 EMT is associated with organ fibrosis, which can lead to organ destruction. Finally, type 3 EMT is associated with migratory and invasive features of tumor cells. A characteristic of type 3 EMT is that it originates from cells that have already undergone malignant transformation. Thus, the genetic and epigenetic changes typical for cancer cells, such as the activation of oncogenes and the inactivation of tumor suppressors, can act in concert with the EMT program.

EMT is considered important at several stages during tumor progression [3]: First, EMT can render tumor cells migratory and invasive, enabling them to detach from their collective and invade through the basal lamina of their tissue of origin (*invasion*); second, it may help gain access to lymph or blood vessels (*intravasation*); third, EMT may play a role for tumor cells to leave the bloodstream again and form micrometastases (*extravasation*). For effective macrometastasis at distant sites, tumor cells must adapt to their new environment, invade into different tissue types and gain strong proliferation ability. During these processes, tumor cells may have to undergo MET, *i.e.*, to change back from a mesenchymal to an epithelial state [4]. Many steps of invasion and metastasis, however, are still poorly understood, and the precise contribution of EMT remains controversial. A critical point is that the concept of EMT in tumor progression has been mainly developed in cell culture systems, and that it is unclear to what extent these findings can be transferred to an *in vivo* setting. The situation is complicated by the fact that EMT is inherently difficult to detect *in vivo*, as it may be a transient event limited to the invasive front. Also, epithelial tumors are often surrounded by mesenchymal cells, which may be either tumor cells after EMT or mesenchymal cells of non-tumor origin. Nevertheless, there is increasing evidence for the validity of the concept of EMT in tumor progression [4].

### 1.2. Pancreatic Adenocarcinoma

Pancreatic cancer is the fourth-leading cause of cancer death in the Western world and is characterized by a particularly poor prognosis, with a five-year-survival rate of only 4% [5,6]. Several well-known factors contribute to this outcome, e.g., early invasion and dissemination of tumor cells, late detection, and resistance to chemo- and radiotherapy. Most malignant pancreatic tumors are adenocarcinomas. While the specific cell of origin of the tumor is not definitely known, much insight has been gained into the development of the disease recently. Pancreatic adenocarcinoma proceeds through distinct, histopathologically defined stages, with the pancreatic intraepithelial neoplasia (PanIN, graded in stages I, II, and III) being the most frequent and well understood premalignant lesion. The disease progression is accompanied by genetic alterations. Virtually all tumors have an oncogenic mutation in the *K-RAS* gene, which is considered an initiating pathogenetic step. Loss of the tumor suppressor INK4A is another important genetic hallmark, which occurs in moderately advanced stages. Later events include mutations of p53, and eventually, in more than half of the cases, loss of SMAD4 (DPC, Deleted in pancreatic cancer). It is interesting to note that two pathways with prominent functions in EMT, the RAS and TGFβ signaling pathways, are disturbed in pancreatic cancer: Ras is constitutively activated due to oncogenic mutations, whereas TGFβ signaling, which has an important dual role as a tumor suppressor and tumor promoter, can be enhanced or blunted. Whereas oncogenic *RAS* mutations and enhanced TGFβ signaling are consistent with EMT and an invasive phenotype, loss of SMAD4 protein, a vital component of canonical TGFβ signaling, is not [7].

## 2. EMT Signaling Pathways

### 2.1. Principles of EMT Signaling

EMT in tumor progression is governed by a complex network of many signaling pathways. There are, however, some principles that are of general significance.

EMT is triggered by extracellular signals, which can be soluble factors or non-soluble components of the extracellular matrix. Members of the TGFβ family and a range of other growth factors, e.g., members of the FGF, HGF or EGF families, are prominent among the soluble factors, whereas collagen and hyaluronic acid are important non-soluble EMT inducers [8]. Accordingly, the receptors involved in EMT signaling include TGFβ receptors, receptor tyrosine kinases, and receptors for ECM components. Within the cell, the signal is relayed via several signaling pathways, e.g., SMAD signaling and small GTPases like RAS. Eventually, transcriptional regulators of the Snail, ZEB, and bHLH families are up-regulated and/or activated. A common feature of these regulators is the repression of the gene that encodes E-cadherin, the principal epithelial adhesion protein [9].

### 2.2. Transforming Growth Factor-β (TGF-β) and SMAD Signaling

TGFβ is arguably the most important EMT-inducing soluble factor in a diverse range of tumor cells. TGFβ is an important regulator of tissue homeostasis with two distinct functions in tumor development and progression [10,11]. In early tumor development, TGFβ has an inhibitory effect on tumor growth. This function is mediated by the inhibition of MYC and ID transcription factors and the induction of cell cycle inhibitors (e.g., p15 and p21), which leads to growth arrest. TGFβ can also induce apoptosis in many cell types [12]. While TGFβ can thus initially act as a tumor suppressor [13], in later disease stages tumor cells become increasingly resistant to the cytostatic effects of TGFβ, and it can in contrast even function as a tumor promoter. This detrimental function may be explained by the fact that TGFβ is one of the most prominent EMT inductors: When treated with TGFβ, many pancreatic carcinoma cell lines show a morphological alteration typical for EMT, lose epithelial and gain mesenchymal markers [14,15,16].

Several pathways mediate the effects of TGFβ and are also central for the biology of pancreatic carcinoma. Binding of TGFβ to a TGFβ type II receptor (TβRII) leads directly or indirectly to the transactivation of a type I receptor (TβRI, or activin receptor-like kinase, ALK). TβRI, a serine/threonine kinase, subsequently phosphorylates SMAD2 and SMAD3, which form heterotrimers with SMAD4 and translocate to the nucleus, where they interact with DNA-binding transcription factors and either activate or repress transcription. TGFβ signaling in EMT is complicated by the fact that the type I receptors ALK1, ALK2, and ALK5 have distinct and sometimes opposing functions: Whereas, for example, the principal ALK5 receptor mediates EMT, ALK1 can counteract this effect [17]. Moreover, the TGFβ signal can be relayed in a non-canonical, *i.e.*, SMAD-independent pathway, which may involve ERK/ MAPK, PI3K, p38, JNK, RhoA, and other signaling pathways [18].

Mutations in the TGFβ pathway observed in pancreatic cancer principally concern SMAD4: Its inactivation is observed in about 55% of cases and is associated with poor prognosis [19,20]. Much rarer are mutations of the TGFβ receptors [5]. All these mutations confer a growth advantage to the tumor cells by preventing the cytostatic effect of TGFβ. However, at the same time, all three TGFβ isoforms are overexpressed and correlate with decreased survival in pancreatic carcinoma, which could be due to increased EMT [21,22]. The distinct activation states of TGFβ signaling in pancreatic carcinoma may thus be a reflection of the fact that TGFβ signaling itself is a double-edged sword, conferring advantages and disadvantages to the tumor.

TGFβ-induced EMT is dependent on several other pathways. One such pathway is the MAPK/ ERK pathway: Treatment of cells with the MEK1 inhibitor PD98059, or expression of mutants of RAS, which is upstream of MAPK/ ERK, can prevent EMT [14,23,24]. Crosstalk between SMAD and STAT3 signaling may also be important for TGFβ-induced EMT [25], with the STAT3 downstream target LIV-1 being a potential mediator [26]. Moreover, TGFβ-induced EMT may also require activation of the transcription factors SP-1 and NF-κB [23,27].

Whether TGFβ-induced EMT is dependent on functional SMAD4 is still a matter of debate due to conflicting experimental data. SMAD4-dependence is supported by the fact that TGFβ cannot induce EMT in the pancreatic cancer cell lines IMIM PC-2 or CAPAN-1, which do not express functional SMAD4 [14]. Moreover, Schniewind *et al*. [28] demonstrated in PANC-1 cells that the SMAD-activating functions of type I TGFβ receptor ALK5 is necessary for both the cytostatic functions of TGFβ and the promotion of EMT and metastasis. However, there are also reports that TGFβ can induce features of EMT independently of SMAD4, and also related alterations such as migration [25,29,30,31]. In line with that, Levy *et al*. [32] suggested that there are two classes of TGFβ-regulated genes: SMAD-dependent, which are important for anti-proliferative effects, and SMAD-independent, which regulate EMT.

Consistent with their function as tumor suppressors, deletion of SMAD4 or TGFβRII in the pancreatic epithelium in mice accelerates KRAS^G12D^-initiated tumors [33,34]. Likewise, SMAD4 deletion accelerated pancreatic adenocarcinoma (PDAC) development in pancreas epithelium expressing a KRAS^G12D^ allele and heterozygous for INK4A/ARF [34]. Most importantly, tumors in these mice differed in their epithelial-mesenchymal differentiation status, with SMAD4-deficient tumors retaining their epithelial differentiation. In cell lines isolated from these mice it could also be shown that most effects of TGFβ were SMAD4-dependent. In addition to SMAD mutations, overexpression of the SMAD-interacting protein Ski can confer resistance to TGFβ-induced cytostatic effects. When Ski is down-regulated by siRNA in pancreatic cancer cells, these cells show decreased proliferation, but at the same time also increased EMT and invasive and metastatic features. Interestingly, this seems to be independent of the SMAD4 status [35].

**Figure 2:**
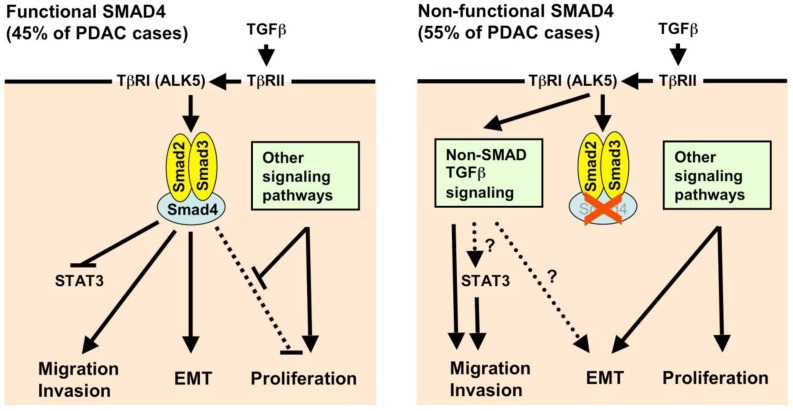
Characteristics of EMT in pancreatic adenocarcinoma (PDAC) with functional or non-functional SMAD4 protein. TGFβ binds to a type 2 TGFβ receptor (TβRII), which phosphorylates TβRI (ALK5). TβRI (ALK5) in turn phosphorylates the receptor-activated SMADs (SMAD2 and SMAD3). **Left**: In tumor cells with functional SMAD4, SMAD2 and SMAD3 form heterotrimeric complexes with SMAD4, translocate to the nucleus and, together with cofactors, alter gene expression. This can lead to EMT, migration, and invasion. The SMAD-induced inhibition of proliferation, which is mediated e.g., by inhibition of Myc and Id factors and activation of p15 and p21, has to be overcome in tumors. This could possibly be achieved by alterations in SMAD target genes, e.g., by alterations in co-activators and co-repressors, or by activation of other signaling pathways which favor proliferation. **Right**: In SMAD-deficient cells, the anti-proliferative TGFβ effect is abolished and thereby confers a growth advantage to these cells. However, SMAD-mediated EMT, migration and invasion is also halted. In that case, non-canonical TGFβ signaling (e.g., MAPK, PI3K, RhoA, PAR6) is believed to mediate migration and invasion. Also, the absence of SMAD4 leads to a disinhibition of STAT3, which mediates migration and invasion. However, whether non-canonical TGFβ signaling actually can induce EMT remains controversial; possibly, other signaling pathways (e.g., NF-κB) are necessary to replace SMAD signaling. Even in that case, the EMT in SMAD4-deficient tumor cells may not be as pronounced as in SMAD4-containing tumors.

Given the conflicting data, it is difficult to draw a general conclusion on the function of TGFβ and SMAD signaling in pancreatic carcinoma. For the tumor, the advantages of inactivated TGFβ signaling obviously outweigh the disadvantages, since SMAD4-deficiency is associated with a poor prognosis [19,20]. Given these data from human patients and the insight gained from mouse models [34], it is likely that the SMAD4 status defines two subclasses of pancreatic carcinoma with very distinct behavior. In tumors with functional SMAD4, the cytostatic effects of TGFβ have to be prevented by other mechanisms (e.g., hyperactive Ras signaling, overexpression of Ski, *etc*.), and thus, TGFβ can confer EMT and the ability to metastasize. SMAD4-deficient tumors, in contrast, have a growth advantage because the cytostatic effects of TGFβ are prevented in the first place. For tumor progression, however, other signaling pathways have to compensate for the loss of canonical TGFβ signaling, be it non-canonical TGFβ signaling or completely different pathways (Figure 2). Whereas in mouse models, there is a correlation between SMAD4 status and histological differentiation/ EMT, it remains unclear whether this is also true for human tumors [5,34].

### 2.3. Other Soluble Factors and Their Signaling Pathways

In addition to TGFβ, other soluble factors have been reported to contribute to EMT in pancreatic carcinoma. Some of them are members of the TGFβ superfamily (BMPs), some are growth factors that exert their function via receptor tyrosine kinases (HGF, VEGF), and a third group signals via other pathways (TNFα, periostin). They may be produced by the tumor cells themselves or by stroma cells (e.g., pancreatic stellate cells), which are very prominent in pancreatic carcinoma.

Bone morphogenetic proteins (BMPs) are members of the TGFβ superfamily and signal via SMAD proteins. They contribute to EMT during development and are now also emerging as important regulators of tumor-associated EMT, migration, and invasion. BMP-2, BMP-4 and BMP-7 can induce EMT in PANC-1 cells [36,37]. This EMT depends on SMAD1 and on the loss of TβRIII and is characterized by increased activity of MMP-2 [37]. Loss of TβRIII had previously been show to be associated with increased motility and invasiveness, albeit without affecting features of EMT [38]. A downstream mediator of BMP-4-induced EMT may be the homeobox gene product MSX2 [36], which by itself enhances migration and metastasis possibly by inducing expression of Twist, a transcription factor with repressive function on the E-cadherin promoter [39].

Tyrosine kinase receptors, when activated by their growth factor ligands, can mediate EMT. In pancreatic carcinoma, examples include c-Met, the receptor for hepatocyte growth factor [40], RON [41], Axl [42], and VEGFR-1, the receptor for VEGF-A and VEGF-B [43]. Knockdown of Axl, e.g., leads to the down-regulation of the EMT effectors Snail, Slug, Twist, and Mmp-9. Another example is vascular endothelial growth factor receptor-1 (VEGFR-1, Flt-1), whose activation by VEGF-A or VEGF-B leads to increased migration, invasion, and EMT in the pancreatic carcinoma cell line L3.6pl [43]. TNFα can stimulate EMT and motility in pancreatic carcinoma cells, even if these lack SMAD4 [23]. TNFα may lead to enhanced expression of the EMT-promoting transcription factor Snail, a process which requires NF-κB activation at least for tumor cell survival [23,44]. The secretory protein periostin (osteoblast-specific factor 2) is strongly expressed in pancreatic cancer. The principal source is probably pancreatic stellate cells and to a lesser extent the tumor cells themselves. The data on the function of periostin in pancreatic cancer are unclear and conflicting. It is likely that it promotes invasion by creating the fibrous environment typical for pancreatic adenocarcinoma, but in one study it was also suspected to promote MET of tumor cells [45,46,47,48].

### 2.4. RAS Signaling

In addition to its role as a regulator of cell differentiation, proliferation, and survival, the GTP-binding protein RAS has been identified as a pre-requisite for EMT in many types of cancer and model systems of EMT. RAS is active in many tumors due to stimulation by receptor tyrosine kinases, or due to an oncogenic mutation in one of the Ras genes. Virtually all cases of pancreatic adenocarcinoma contain oncogenically mutated, constitutively active K-RAS [7]. RAS can activate several downstream pathways, including the RAF-MAPK, the PI3K, and RalGDS pathways. The RAF-MAPK pathway appears to be the one necessary for EMT [49].

It has been known for some time that TGFβ-induced EMT of pancreatic carcinoma cells is dependent on activation of RAS/RAF/MEK/ERK signaling [14], but even activation of RAS alone can have an effect on EMT: The introduction of oncogenic K-RAS (V12) in primary pancreatic duct epithelial cells not only drives cell cycle progression and cell growth, but also leads to loss of E-cadherin and gain of N-cadherin expression [50]. *In vivo*, it was demonstrated that expression of oncogenic K-RAS in the pancreas is sufficient to induce precursor lesions and invasive pancreatic carcinoma similar to human disease [51]. While this study did not directly address the effect of K-RAS on the epithelial-mesenchymal cell state, Brembeck *et al*. [52] showed that transgenic expression of oncogenic K-RAS (V12) under the cytokeratin 19 promoter led to an enhanced expression of the mesenchymal marker N-cadherin in pancreatic ductal cells. It should be noted, however, that the effect of K-RAS on EMT probably depends largely on context, since activating *K-RAS* mutations can also be found in normal epithelial tissue adjacent to tumors [53].

In a study analyzing K-RAS dependency by RNA interference, *K-RAS*-mutant cell lines—among them pancreatic carcinoma cell lines—could be divided into two classes: those which were dependent on RAS for survival ("oncogene-addicted"), and those which were not [54]. Interestingly, there was a link between K-RAS-dependency and epithelial differentiation status: Cells addicted to K-RAS exhibited a classical epithelial morphology with strong cortical E-cadherin - in contrast to cells no longer dependent on K-RAS, which appeared less epithelial, had lost expression of E-cadherin and instead showed expression of vimentin and the EMT-promoting transcription factor ZEB1. Ablation of ZEB1 expression in PANC-1 cells led to an increase in K-RAS-dependency, suggesting that epithelial-mesenchymal state and K-RAS dependency are interconnected [54].

### 2.5. Transcriptional Repressors: Snail, ZEB, and bHLH Families

The contribution of transcriptional repressors of the ZEB, Snail, and bHLH (basic helix-loop-helix) families to EMT have been extensively studied in recent years [9].

ZEB1 (δEF1) and ZEB2 (SIP1, for SMAD-interacting protein) are zinc finger transcription factors encoded by two separate genes (ZFHX1A and ZFHX1B, [9]). ZEB1 has been identified as a direct transcriptional repressor of E-cadherin [55]. In pancreatic carcinoma, ZEB1 can be up-regulated by IKK/ NF-κB activation [23]. Moreover, it has been shown recently to be part of a feed-forward loop involving members of the microRNA-200 family: ZEB1 can repress the microRNA-200 family members miR-141 and miR-200c, which in turn are repressors of the EMT promoters TGFβ2 and ZEB1 [56]. The role of ZEB1, however, is not limited to EMT promotion; it obviously also acts as a promoter of stemness, again by inhibition of microRNAs [57,58]. It is likely that ZEB2 also plays a role in EMT of pancreatic carcinoma cells, for ZEB2 mRNA levels inversely correlate with the expression of E-cadherin and increase with advanced tumor stages [59]. Both ZEB1 and ZEB2 are subject to regulation by co-factors, which can be activating or repressive [9].

Among the basic helix-loop-helix transcription factors important for EMT are E47 (encoded by the E2A gene) and Twist. Like the Snail superfamily of zinc-finger transcriptional repressors, which comprises Snail (SNAI1) and Slug (SNAI2), they have a repressive effect on the E-cadherin (*CDH1*) gene and also regulate several other EMT-related genes [9]. In pancreatic cancer, snail expression correlates with that of integrin-linked kinase (ILK), a known inducer of EMT [60]. Ectopic expression of Snail is sufficient to promote EMT, invasion, and metastasis in the pancreatic cancer cell line BxPC3 [61]. While most of the mechanistic understanding of these factors originates from other tumor entities, there is evidence that they also play a pivotal role in pancreatic carcinoma [62,63,64,65].

### 2.6. Other Signaling Pathways

NF-κB signaling has long been associated with tumorigenesis because of its anti-apoptotic effect in many types of cancer cells. Later, NF-κB was identified as a central regulator of EMT in mammary epithelial cells [66,67]. In pancreatic carcinoma, it can control metastasis and EMT: Fujioka *et al*. [68] inhibited NF-κB in pancreatic cancer cells (AsPc-1) by expressing an IκBα superrepressor, which led to decreased metastasis in nude mice upon orthotopic injection of tumor cells. Mechanistically, this finding was explained by a decrease in tumor angiogenesis, because the up-regulation of VEGF, a target gene of NF-κB, was inhibited. Another potential mechanism, however, could be the inhibition of EMT. It has been shown that pancreatic cancer cells expressing the IκBα superrepressor do not undergo TGFβ-induced EMT [23]. On the other hand, hyperactivation of NF-κB by expression of a constitutively active form of its upstream kinase IKK2 (CA-IKK2) is sufficient to induce EMT, even in the absence of a functional TGFβ signaling pathway. At least in part, this occurs independently of RAS signaling [23]. In mammary carcinoma, the importance of this pathway was demonstrated by the application of a low molecular weight inhibitor of IKK2, which not only prevented EMT induction by CA-IKK2, but also attenuated metastasis from orthotopic sites induced by 4T1 cells [69]. These findings could explain why loss of SMAD4 does not prevent tumor progression, since there are alternative pathways which can replace TGFβ signaling. Mechanistically, this function of NF-κB could be due, at least in part, to the up-regulation of ZEB1, which is a target gene of NF-κB [23,70].

Hedgehog signaling is acknowledged as an important factor in the development of the gastrointestinal tract and has been associated with pancreatic cancer invasiveness and metastasis. The pathway has received considerable attention because inhibitors of hedgehog signaling are available and could prove interesting for clinical application [71]. In part, the effect of hedgehog could be due to a modulation of EMT: Feldmann *et al*. [72] showed that blockade of hedgehog with cyclopamine reversed markers of EMT like E-cadherin and snail in pancreatic cancer cell lines. Bioinformatic studies suggest that the hedgehog pathway could signal via TGFβ to ZEB2 [73].

Wnt signaling is linked to EMT via the intracellular domain of the cadherins, which can activate β-catenin/wnt signaling [74]. In addition, several other connections have been proposed. For example, pancreatic cancer cells undergo a MET when exposed to recombinant WISP-2/CCN5, suggesting that the protein is required for maintaining an epithelial state [75]. Interestingly, WISP-2/CCN5, a member of the WNT1-inducible signaling pathway (WISP), is down-regulated in pancreatic adenocarcinoma and in cell lines from pancreatic carcinoma. In contrast, WNT5A, a secreted member of the WNT family, seems to be overexpressed in pancreatic carcinoma and is associated with features of migration, invasion, and EMT. It can be up-regulated by the transcription factor CUTL1, an important mediator of the invasive and metastatic effects of TGFβ in pancreatic carcinoma [76].

Still other factors have been associated with EMT in pancreatic carcinoma. For example, Notch signaling has been implicated in EMT because its down-regulation by siRNA leads to partial MET. Interestingly, Notch-2 and its ligand Jagged-1 are up-regulated in gemcitabine-resistant cells, providing a link to chemoresistance [77]. A very recent study provided evidence that the loss of another transcription factor, FOXA1/2, is essential for EMT in pancreatic carcinoma [78].

### 2.7. Cadherins

Many of the EMT pathways described so far eventually lead to a switch in the expression of cadherins, a large family of type 1 transmembrane proteins that mediate cell-cell adhesion. During EMT, cells often undergo an isoform switch from E-cadherin to N-cadherin [79]. Both isoforms are thus commonly used as marker proteins for the determination of the epithelial/ mesenchymal status of a cell. E-cadherin is considered the principal epithelial adhesion protein, whereas N-cadherin is not normally expressed in epithelial cells. Via their intracellular domains, cadherins are not only linked to the actin cytoskeleton, but also to a range of signaling pathways, particularly Wnt/β-catenin signaling [74]. It is increasingly recognized that a change in the expression of cadherin isoforms not only accompanies EMT, but can also play a causal role in its initiation or promotion.

E-cadherin is frequently lost in carcinomas due to inactivating mutations or transcriptional repression. It has been shown that this loss is not just an epiphenomenon of tumor progression, but can by itself be causal for the progression of pancreatic tumors [80]. Moreover, the re-expression of E-cadherin in E-cadherin-deficient pancreatic carcinoma cells leads to MET and a decrease in invasive potential [81]. This holds true also in an *in vivo* mouse model, where genetic inactivation of E-cadherin induces EMT and promotes metastasis *in vivo* [82]. The isoform N-cadherin, in contrast, is considered a promoter of invasion and metastasis; its expression correlates with histological grade and neural invasion in pancreatic tumors, for example [81,83]. Migration and invasion is also enhanced by polysialylated neural cell adhesion molecule (PSA-NCAM), which can reduce E-cadherin-mediated cell-cell adhesion [84].

### 2.8. The Extracellular Matrix and Proteases

Pancreatic carcinoma is characterized by excessive extracellular matrix deposition, which strongly influences signaling in the tumor cells [85,86]. Collagen-I is a main component of these deposits and can decrease E-cadherin and increase N-cadherin expression, disrupt E-cadherin-mediated cell-cell contacts, and stimulate migration and metastasis of pancreatic carcinoma cells [87,88,89,90]. The effects of collagen on N-cadherin expression seem to be mediated by α2β1 integrin and discoidin domain receptor 1, whereas the effects of N-cadherin on cell behavior requires JNK activation [89,91].

Matrix metalloproteinases (MMPs) are overexpressed in almost all types of tumors. They have an important role for invasion and metastasis because they enable the digestion and remodeling of the extracellular matrix, and are increasingly recognized to influence also cell signaling and EMT [92]. In addition to MMPs, other proteases have been shown to influence EMT. HAI-1 (Hepatocyte growth factor activator inhibitor-1) is a membrane-associated protease inhibitor which can stabilize the epithelial phenotype and whose knockdown leads to EMT and up-regulation of SIP1 and MMP-9. It exerts its function by the inhibition of membrane-bound serine proteases, e.g., TMPRSS4 [93].

A target of proteases is heparin-binding epidermal growth factor-like growth factor (HB-EGF), which exists in an uncleaved membrane-bound and in a cleaved soluble form. Wang *et al*. [94] showed that the expression of the uncleaved form (pro-HB-EGF) on the membrane of pancreatic cancer cells stabilized E-cadherin and thus the epithelial phenotype by inhibiting ZEB1.

### 2.9. MicroRNAs and Epigenetic Mechanisms

MicroRNAs are emerging as another level of control of the epithelial-mesenchymal state of tumor cells. Several microRNAs have been suggested to be involved in EMT [95]. Members of the miR-200 family have been convincingly associated with EMT in pancreatic cancer [56,96,97]. There is evidence for an intricate network of microRNAs and the transcription factor ZEB1, which may not only regulate EMT, but also provide links to stem cell-like properties and chemoresistance of tumor cells [57,58,98].

Epigenetic mechanisms, e.g., promoter regulation through DNA methylation or histone modification, have long been acknowledged as important tumor-promoting events. The high-mobility group A2 (HMGA2) protein, a non-histone chromatin factor important for mesenchymal differentiation, is overexpressed in pancreatic carcinoma [99] and was proposed to maintain a mesenchymal state of advanced pancreatic carcinoma cells by activating the Snail promoter [100]. Histone deacetylases, which lead to chromatin silencing by removing acetyl groups from lysyl residues of histone proteins, have been highlighted in a recent work by von Burstin *et al*. [82]. The authors suggest that Snail can suppress the transcription of the prototypic epithelial adhesion protein E-cadherin by forming a complex with HDAC1 and HDAC2.

## 3. Evidence for EMT in Human Tissue Samples of Pancreatic Carcinoma

The principles of EMT have been discovered in cell culture models, which are easy to handle and allow for fast genetic modification by overexpression or knockdown of respective proteins. However, it is uncertain to what extent these cell culture models faithfully reflect the *in vivo* situation in complex human tumor tissues. Furthermore, EMT may only occur at the invasive front of tumors only, and is likely to be a transient event. This problem has led some researchers to believe that the whole concept of EMT in tumor progression is invalid [101,102]. In human tumor samples stained with hematoxylin and eosin, EMT is often undetectable because cells after EMT fail to show a spindle- or fibroblastic morphology in these tumor tissues. EMT can be detected, however, in such samples by staining for EMT markers [103], which is not done in standard clinical practice.

Recent studies in breast carcinoma—showing a correlation between the expression of EMT markers in tumor tissue samples and histopathological or clinical parameters [104,105,106,107]—encouraged investigations for EMT markers in other tumors. Indeed, respective observations were made in a range of very diverse tumor entities, such as in lung, nasopharyngeal, esophageal, ovarian, endometrial, and prostatic carcinomas. Common established markers of EMT are loss of E-cadherin and gain of N-cadherin and vimentin expression, as well as an increased expression of the EMT-inducing transcription factors Snail, Slug, Twist, ZEB1, and ZEB2.

There is now evidence for EMT in several tumor entities of the gastrointestinal tract [108]. In pancreatic carcinoma, however, studies have come to contradictory results. Javle *et al*. [109] saw a correlation between EMT markers (high fibronectin and vimentin, low E-cadherin) in 36 surgically resected pancreatic carcinoma samples and poor survival. In line with this, it was shown that down-regulation of E-cadherin might be a marker suitable for the prediction of metastasis in pancreatic cancer, albeit only in combination with up-regulation of urokinase-type plasminogen activator (uPA) [110]. In another study on primary pancreatic carcinoma, N-cadherin, which is not expressed in normal pancreatic tissue, was detected in 13 out of 30 samples [83]. There was also a correlation between N-cadherin expression and neural invasion or histological type. Vimentin was increased in metastases as compared to primary tumors. However, no inverse correlation between E-cadherin and N-cadherin expression could be detected, *i.e.*, a cadherin switch, as the one observed in prostate cancer [111] did not take place. In contrast to this study, Cates *et al*. [63] could not detect N-cadherin in tissue samples of pancreatic carcinoma.

The transcriptional repressor Snail was expressed in 36% to 78% of pancreatic cancer tissue samples and may or may not correlate with tumor stage, lymph nodal status and distant metastasis, depending on the study [62,64]. It is of note that undifferentiated tumor cell lines (MiaPaCa-2, Panc-1) tend to have a higher Snail expression than differentiated ones (Capan-1, HPAF-2, AsPC-1) [64].

Slug expression was reported to be increased in pancreatic carcinoma as compared to normal surrounding tissue in one study [64], whereas another study could not detect any differences in expression between pancreatic carcinoma, chronic pancreatitis, and normal pancreas [63]. In any case, no correlations between Slug expression and relevant clinical data could be established. Data from pancreatic cancer cell lines also suggest that there is no correlation between Slug expression and grade of dedifferentiation [64].

The available data on the expression of Twist in pancreatic carcinoma are contradictory: one study reported an up-regulation of Twist in cancer, another a down-regulation of nuclear Twist, and a third no expression at all in tissue samples [63,64,65]. Interestingly, Twist expression can be induced in pancreatic cancer cell lines by hypoxia [64], a condition which can also induce EMT [112].

According to a recent study, ZEB1 is not expressed in normal pancreatic tissue and only weakly in well-differentiated pancreatic carcinoma, whereas it is strongly expressed in dedifferentiated pancreatic carcinoma. Reduced ZEB1 expression correlated significantly with lack of tumor recurrence after complete resection [58].

In summary, the data on EMT markers in pancreatic carcinoma are not as clear-cut as in some other tumors and actually are in part contradictory, which may be due to heterogeneous patient populations, small sample numbers, or different methodological approaches. One should also keep in mind that detection of EMT may be difficult, since it may occur in some cells at the invasive front only (which can then be misidentified as stroma cells if not co-stained for cytokeratins 8/18), or since cells after EMT might soon undergo the reverse process [113]. Furthermore, EMT may comprise multiple phenotypes, from weak or partial to complete expression of the multiple morphological and protein marker characteristics of EMT.

## 4. EMT, Hypoxia, Inflammation, Invasion, and Metastasis

Rapid tumor growth frequently leads to hypoxia, which in turn influences signaling events within the tumor cell and eventually leads to the activation of an angiogenic program and EMT. In tumor cell lines, among them PANC-1 cells, EMT induced by hypoxia is accompanied by snail nuclear translocation, inhibition of GSK3β, and later on by activation of Wnt/β-catenin signaling [112]. It is likely that the transition is regulated via activation of Twist via HIF-1α, as has been shown in other cell lines [114].

Inflammation is important during different stages of tumor development and progression [115]. There are numerous potential connections between inflammation and EMT, which need to be evaluated experimentally. One important link is the transcription factor NF-κB, which mediates both inflammation and EMT. Another connection could be the pro-inflammatory cytokine TNFα, which can also induce an EMT-like phenotype. Different chemokines released during chronic inflammation can also play important roles in EMT (A. Gal and HB, unpublished). TGFβ, in contrast, has an anti-inflammatory effect [116].

An association between EMT and invasion/metastasis has been made in numerous studies. For example, an EMT program is induced when highly metastatic pancreatic cancer cells are selected *in vivo* [82]. Besides E-cadherin, ZEB1 seems to be an important link between EMT and invasion [56]. Moreover, it was shown that the number of solitary infiltrating pancreatic carcinoma cells is an independent adverse prognostic factor and correlates with EMT markers such as decreased E-cadherin and increased vimentin expression [117]. However, it should be noted that invasion can in principle also occur in the absence of EMT [118]. It was also proposed recently that EMT may be only important for providing a microenvironment facilitating invasion, but that the invading cells are indeed cells which have not undergone EMT [119]. Data from other tumor entities, however, suggest that EMT actually is a hallmark of invading cells. For instance, cells at the invasion front of colon carcinomas showed a clear EMT phenotype [103]. Another recent example is that the reversion of EMT in MDA-MB-231 cells by re-expression of annexin A1 (lack of which is a strong predictive factor for metastasis and impeded survival in human breast cancer tissue arrays) also abolished the strong metastatic potential of these cells [120].

## 5. EMT and Cancer Stem Cells

It is a common clinical observation that individual tumor cells in a patient respond differentially towards chemotherapy. Often, just a small (and frequently undetectable) fraction of the tumor cells is resistant to chemotherapy, but these cells have the ability to give rise to a new tumor. Experimental work has demonstrated that individual tumor cells of a single tumor have different tumorigenic potential when injected into recipient mice. Thus, tumors are heterogeneous tissues composed of cells with distinct properties. An important concept for explaining the described clinical and experimental observations is the cancer stem cell hypothesis, which states that a subpopulation of the cells within a tumor has the ability to self-renew, to seed tumors in recipient animals, and to give rise to differentiated progeny [121].

EMT is commonly understood as a transdifferentiation rather than a dedifferentiation process. That is why it came as a surprise when Mani *et al*. demonstrated in mammary epithelial cells that EMT also conferred stem cell properties to tumor cells and that stem cells exhibited features of EMT [122,123,124]. A closely related concept is that of migrating cancer stem cells [125,126]. In pancreatic carcinoma, the link between EMT and cancer stem cells has also been met with great interest [127,128].

In the pancreas, several approaches have been taken to isolate cancer stem cells. Hermann *et al*. defined two distinct populations, one of which was characterized by high tumorigenicity and chemoresistance (CD133+), whereas the other was characterized by its migratory and metastatic potential [129]. Li *et al*. isolated a different subpopulation with stem-like properties, which showed increased expression of sonic hedgehog [130]. However, these cell populations were not assessed for EMT markers.

It is also possible to purify cells with stem-like properties out of existing cell lines. For example, "side population" stem cells can be purified based on the efflux of fluorescent dyes, which is a correlate of the expression of a specific ABC transporter [131]. Kabashima *et al*. [132] characterized side population cells purified from pancreatic carcinoma cell lines. Surprisingly, side population cells had a strong epithelial phenotype with robust E-cadherin expression; however, they underwent TGFβ-induced EMT more readily than cells of the main population, and manifested an increased invasive and metastatic potential. In another study, pancreatic cancer cells were divided into a fast- and slow-cycling population based on their ability to retain DiI label [133]. In contrast to the previous study, the slow-cycling stem cell population manifested morphological and molecular alterations reminiscent of EMT in the unstimulated state. However, the isolation of cells with stem cell-like properties out of existing cell lines remains problematic.

Wellner *et al*. [58] provided the perhaps most convincing explanation of the possible link between EMT and stemness, as they could show that the transcription factor ZEB1 not only promotes EMT, but at the same time confers stem-like properties by inhibiting microRNAs of the MiR-200 family.

## 6. EMT and Drug Resistance

Since only a small proportion of patients diagnosed with pancreatic carcinoma are eligible for curative surgery, chemotherapy could be—at least theoretically—an attractive option in managing the disease. However, a major problem preventing success is drug resistance, which is often acquired during treatment. It has been known for some time that there are connections between EMT and drug resistance [127,134]. Three observations support this notion: (i) Cells which have undergone EMT tend to be more resistant to drugs; (ii) When cells are cultured in the presence of a certain drug, specific drug-resistant cells will have a selective advantage; these cells will often have undergone EMT; (iii) Targeting EMT pathways not only leads to a decrease in invasive potential, but also to an increase in drug sensitivity.

The nucleoside analog gemcitabine is used for chemotherapy of pancreatic cancer either alone or in combination, e.g., with the nucleoside analog 5-fluorouracil or erlotinib, a small molecule inhibitor of EGFR [6]. Most of the studies addressing chemoresistance in pancreatic carcinoma rely on gemcitabine or erlotinib treatment of established pancreatic cancer cell lines. When L3.6pl and AsPC-1 cells were cultured in serially increasing concentrations of gemcitabine, they gained not only an up to 50-fold increase in drug resistance, but also showed hallmarks of EMT [135]. These included morphological alterations, e.g., a change to a spindle-shaped morphology, as well as functional alterations, e.g., increased migratory and invasive potential, and molecular alterations, including up-regulation of vimentin and down-regulation of E-cadherin. Moreover, gene expression profiling of pancreatic cancer cell lines revealed that cells resistant or sensitive to three chemotherapeutic agents (gemcitabine, 5-fluorouracil, and cisplatin) formed two distinct groups, with features of EMT in the drug-resistant group [136].

Mechanistically, ZEB1 is involved in chemoresistance, as silencing of ZEB1 can restore drug sensitivity in drug-resistant cells [136]. In addition, microRNA profiling revealed down-regulation of microRNAs (e.g., miR-200 family) in gemcitabine-resistant cells. Re-expression of miR-200 by transfection or treatment with natural agents led to a down-regulation of ZEB1 and an increased drug sensitivity [98]. The activation of Notch signaling, which had previously been associated with EMT, may play an important role in drug resistance against gemcitabine, because down-regulation of Notch by siRNA leads to partial MET [77]. Gemcitabine sensitization can also be achieved by inhibition of the EMT regulator LOXL2 [137]. In contrast, overexpression of the homeobox gene product MSX2, a known inducer of Twist, can elicit chemoresistance in pancreatic cancer cells [138].

EMT limits the sensitivity of pancreatic carcinoma cells not only to gemcitabine, but also to drugs directed against the epidermal growth factor receptor, for example erlotinib [139,140]. A specific isoform of hMena, a member of the enabled/vasodilator-stimulated phosphoprotein family, correlates with chemosensitivity for the EGFR inhibitor erlotinib and an epithelial phenotype [141]. When Wang *et al*. expressed pro-HB-EGF on the membrane of pancreatic cancer cells and thereby stabilized the epithelial phenotype, the cells became at the same time more sensitive towards treatment with gemcitabine and erlotinib. This might also be due to inhibition of ZEB1 [94].

## 7. Conclusions and Outlook

As in many other tumors, EMT is emerging as an important event in the progression of pancreatic carcinoma, although its precise contribution remains unclear. A central pathway for EMT in pancreatic cancer is TGFβ signaling. However, its dual role as both a tumor suppressor and promoter has not yet been completely understood. A very promising discovery is the link between EMT, cancer stem cells and chemoresistance. A better understanding of the role of ZEB proteins and micro-RNAs in this context may provide new therapeutic options.

“Differentiation therapy”, *i.e.*, the restoration of a polarized epithelial phenotype in cells that have undergone EMT, is emerging as an exciting novel therapeutic approach in oncology. A pharmacological compound of interest in this context could be salinomycin. This drug selectively targets cells with properties of cancer stem cells, leaving the bulk of cancer cells unaffected [142,143], and was recently found to reverse EMT in several epithelial cell models [120].

A rapidly increasing body of literature supports a related concept. EMT involves a shift from the apical-basolateral polarity of epithelial cells towards the anterior-posterior (front-rear) polarity of motile, fibroblastoid cells [144]. Both types of polarity are initiated and maintained by multiple cooperating multiprotein complexes which govern domain-specific vesicle trafficking, membrane domain identity and responses of these polarity machines to external cues through receptors and signal transduction pathways. Interestingly, signaling pathways such as STAT3, PI3K, Wnt, Notch and hedgehog also play a role in control of epithelial polarity [3,120]. It is likely that research along these lines will identify a large number of future targets for therapeutic intervention in tumor progression and metastasis of epithelial tumors, including pancreatic adenocarcinoma.
